# Effects of oral of administration of monoglycide laurate on virus load and inflammation in PEDV infected porcine

**DOI:** 10.3389/fvets.2022.980381

**Published:** 2022-10-13

**Authors:** Zheyan Liu, Ling Zhu, Xiaonan Zhao, Jian Liu, Huangzuo Cheng, Lina Zhang, Huaqiao Tang, Xiangang Sun, Youjun Hu, Zhiwen Xu

**Affiliations:** ^1^College of Veterinary Medicine, Sichuan Agricultural University, Chengdu, China; ^2^Key Laboratory of Animal Diseases and Human Health of Sichuan Province, Chengdu, China; ^3^Innovation Center of Guangdong Nuacid Biotechnology Co., Ltd., Qingyuan, China; ^4^College of Animal Science and Technology, Jiangxi Agricultural University, Nanchang, China

**Keywords:** porcine epidemic diarrhea virus, monoglyceride laurate, clinical signs, pathological changes, viral load, cytokines

## Abstract

To investigate the effect of monoglyceryl laurate (GML) against PEDV *in vivo*, the clinical signs, pathological changes, tissue viral load and cytokine levels of piglets were compared in different GML treatment groups and PEDV infected group. The diets of experimental groups were supplemented with different doses of GML (5g for A1, 10g for A2, 20g for A3) on day 1, 2, and 3 after PEDV challenge, and the virus challenge group (group C) and blank group (group B) were set as control. The results showed that compared with group C, groups As could reduce the mortality rate of piglets, among which the protection rates of groups A2 and A3 could reach 100%. The trend of weight loss of piglets was effectively slowed down and growth performance recovered in GML treated groups. GML reduced the pathological damage of intestinal tract and the viral load in intestine and mesenteric lymph nodes. The levels of IL-8 and TNF-α in the blood of group As were inhibited by GML in a dose-dependent manner when compared with group C. Our study suggests that GML has potential anti-PEDV effects *in vivo*.

## Introduction

PEDV is a single-stranded positive-stranded RNA virus with a capsid, a member of the family Coronaviridae and the genus of Alpha Coronavirus, and has been mutating since 2010, with outbreaks in China, USA, Korea and other countries (1–3). PEDV can cause infection in pigs of all ages, mainly in lactating piglets and fattening pigs. Affected piglets often show signs of acute watery diarrhea, vomiting and dehydration, with high mortality rates (4). Surviving piglets have slow growth, reduced feed remuneration and lower economic efficiency. Currently, PEDV is mainly transmitted through direct or indirect contact and aerosols. PEDV mutates rapidly and is difficult to prevent and control with traditional commercial vaccines. Therefore, there is an urgent need to develop a more safe and effective anti-PEDV drugs.

In recent years, the clinical use of bioactive substances with antiviral activity as additives or in combination formulations has provided new ideas for virus diseases control. GML is a synthetic ester of glycerol and lauric acid that is readily absorbed and rapidly metabolized by oxidation (5). It can rapidly supply energy to the animal body. It has antiviral, anti-inflammatory, immunomodulatory and growth enhancing properties in piglets (6). *In vitro* studies have shown that low concentrations of GML can inhibit ASFV activity under liquid conditions, and that high doses of GML can significantly reduce the infectivity of ASFV in feed (7). Zhang (8) found that the addition of GML to the diet could reduce the viremia and immunosuppression in sows with PRRS. In addition, studies have shown that GML has a strong resistance and destructive effect on vesicular viruses, such as herpes simplex virus, influenza virus, etc., both *in vivo* and *in vitro* (9). A recent study found that the addition of a certain percentage of alpha-monolaurin to the feed of sows can cut off vertical transmission of PEDV (10). These results suggest that GML may be a new additive for the prevention and control of PED.

The aim of this study was to evaluated the anti-PEDV effect of GML by studying the clinical signs, pathological changes, viral load and cytokine production in piglets infected with PEDV. Different doses of GML have been used as feed additive to elucidate the protective effects of GML on PEDV infected piglets to provide new ideas for the treatment of this dangerous viral infectious disease.

## Materials and methods

### Materials and reagents

PEDV SC strain was identified and preserved by the Animal Biotechnology Center of Sichuan Agricultural University; Total of 25 piglets were purchased from Chengdu Wangjiang Agricultural and Livestock Technology Co Ltd; GML was prepared by Guangdong Nuacid Biotechnology Co Ltd; PrimeScript™ RT kit (PerfectRealTime), DNA/RNA extraction kit, TB Green^®^ PremixExTaq™ (TliRNaseHPlus) were purchased from Takara (Dalian) Engineering Co Ltd.

### Animals and grouping

Pigs were reared in the experimental condition according to the environment of a large-scale farm to prevent stress and infection with fullness a full-price feed and water. The piglets were observed for 7 days and no abnormalities were found before experiment. The piglets (6-week-old) were divided into 5 groups (*n* = 5). A1, A2 and A3 were the low, medium and high dose treatment groups respectively, B was the blank control group and C was the virus control group. According to the previous experimental experience, the challenge dose of 10TCID50 was selected, and 30 mL of liquid was orally administered to each pig in groups A and C. Group B received 30 mL normal saline orally. On the 1st, 2nd and 3rd day after challenge, 5, 10 and 20 g of GML were added and mixed into the diet of groups A1, A2 and A3, respectively, while groups B and C were given the same weight of normal saline as GML. Detailed information is shown in Table 1.

**Table 1:** Information on piglet grouping, attack and dosing.

**Grouping**	**Number**	**Toxic dose**	**Toxic dose (infusion)**	**Sample No. 1**	**Time point of administration**
A_1_	5	10^5^ TCID_50_	30 mL	5 g	
A_2_	5	10^5^ TCID_50_	30 mL	10 g	
A_3_	5	10^5^ TCID_50_	30 mL	20 g	Days 1, 2 and 3 after the attack
B	5	Virus-free cytosol	30 mL	Sanitary saline	
C	5	10^5^ TCID_50_	30 mL	Sanitary saline	

### Clinical symptoms observation and sample collection

All pigs were examined daily for signs of diarrhea, depression, and anorexia signs. The body weight of each pig was measured at 1 days (6 weeks of age) and once a day thereafter until the end of the study (7 days). The average piglets weight (APW; kilograms) was analyzed at 7th day. Blood was collected from all pigs at 0 and 3 days in 5 ml serum separator tubes. The blood was centrifuged at 2,000 g for 10 min and serum aliquots were stored at −80°C until the cytokine testing. Feces were collected from day 1 to day 7 and dissolved in 1.5-ml centrifuge tubes with PBS. Feces were stored at −80°C until viral load testing.

All pigs were executed on the 7th day of the virus attack and intestinal tissues and mesenteric lymph nodes were immediately collected and stored at −80°C. Some of the intestinal tissues and mesenteric lymph nodes were fixed with 4% paraformaldehyde.

### Histopathological observations

The 4% paraformaldehyde-fixed ileum tissue and mesenteric lymph nodes was dehydrated, permeabilized, embedded, sectioned, dewaxed, rehydrated, stained and sealed, and HE-stained sections were prepared to observe histopathological changes under a light microscope and photographed.

### RT-qPCR quantification of PEDV load

Piglet intestinal tissues, mesenteric lymph nodes and feces were collected, ground and mixed thoroughly with 3.0 mL PBS and centrifuged at 12,000 r/min for 3 min. Total RNA from the small intestine,lymph nodes and feces were extracted using RNA iso Plus (Takara, Dalian, China) reagent. Then cDNA was synthesized using PrimeScript^®^RT reagent kit with gDNA Eraser (Takara, Dalian, China). Real-time quantitative PCR was performed using SYBR^®^ Premix Ex Taq™ (Tli RNaseHPlus) (Takara, Dalian, China). The relative viral load levels were evaluated by detection of ORF1 gene expression of PEDV. The upstream primer for detection of PEDV ORF1 gene was AAATGGGAAGTCGGCAGA, and the downstream primer sequence was GTTTTGTTGTGGCGGTAG. The reaction system is shown in Table 2; the reaction procedure was pre-denaturation at 95°C for 30 s; 95°C for 5 s; 60°C for 30 s; 40 cycles were performed. Melting curve: 95°C, 65°C increasing 0.5°C per second to 95°C.

**Table 2:** RT-qPCR reaction system of PEDV ORF1 gene.

**Primer F**	**0.5 μL**
Primer R	0.5 μL
ddH2O	3 μL
SYBR Green Premix Ex Taq II	5 μL
cDNA	1 μL
Total	10 μL

### Cytokine assay

The systemic cytokine profile (i.e., IFN-α, IFN-γ, IL-1β, IL-6, IL-8, IL-10 and TNF-α) was evaluated in serum samples collected at 0, and 3 days p.i.All cytokine tests were performed according to the kit (Elabscience) instructions.

### Statistical analysis

Results are expressed as mean ± standard deviation (SD). Significant differences were determined using one-way analysis of variance (ANOVA) and multiple samples were analyzed in SPSS 20.0 (IBM Corp., Armonk, NY, USA) using Duncan's method and considered statistically significant at *p* < 0.05. One-way analysis of variance (ANOVA) was conducted with the Graph Pad Prism 5 software; *P* < 0.05 was considered a statistically significant difference.

## Results

### Clinical symptoms

As shown in Table 3, one pig died in group A1 on 3th day after the virus attack. No piglets died in the group A_2_, the group A_3_ and the group B. In the group C, two piglets died on second day, one on the fourth day and the other on the sixth day after infection, and only one piglet survived until 7 d. Two days after PEDV infection, piglets in group A appeared clinical symptoms such as diarrhea, anorexia and depression, and recovered after GML administration. The symptoms were similar in groups A_2_ and A_3_. Group B showed no significant changes.

**Table 3:** Statistics of clinical symptoms in each group after the attack.

**Grouping**	**Symptoms**	**Time after attack (days)**						
		**1**	**2**	**3**	**4**	**5**	**6**	**7**
A1	Diarrhea	0/5	4/5	4/4	3/4	3/4	1/4	0/4
	Depressed spirit	0/5	5/5	4/4	4/4	4/4	4/4	1/4
	Anorexia	0/5	2/5	2/4	2/4	1/4	0/4	0/4
A2	Diarrhea	0/5	4/5	5/5	3/5	3/5	1/5	0/5
	Depressed spirit	0/5	5/5	5/5	5/5	5/5	3/5	3/5
	Anorexia	0/5	3/5	3/5	1/5	1/5	0/5	0/5
A3	Diarrhea	0/5	4/5	5/5	3/5	3/5	1/5	0/5
	Depressed spirit	0/5	5/5	5/5	5/5	5/5	3/5	3/5
	Anorexia	0/5	3/5	2/5	1/5	0/5	0/5	0/5
B	Diarrhea	0/5	0/5	0/5	0/5	0/5	0/5	0/5
	Depressed spirit	0/5	0/5	0/5	0/5	0/5	0/5	0/5
	Anorexia	0/5	0/5	0/5	0/5	0/5	0/5	0/5
C	Diarrhea	0/5	3/5	3/3	2/2	2/2	1/1	1/1
	Depressed spirit	0/5	3/5	3/3	2/2	2/2	1/1	1/1
	Anorexia	0/5	3/5	3/3	2/2	2/2	1/1	1/1

### Change in piglet weight

**Figure 1:**
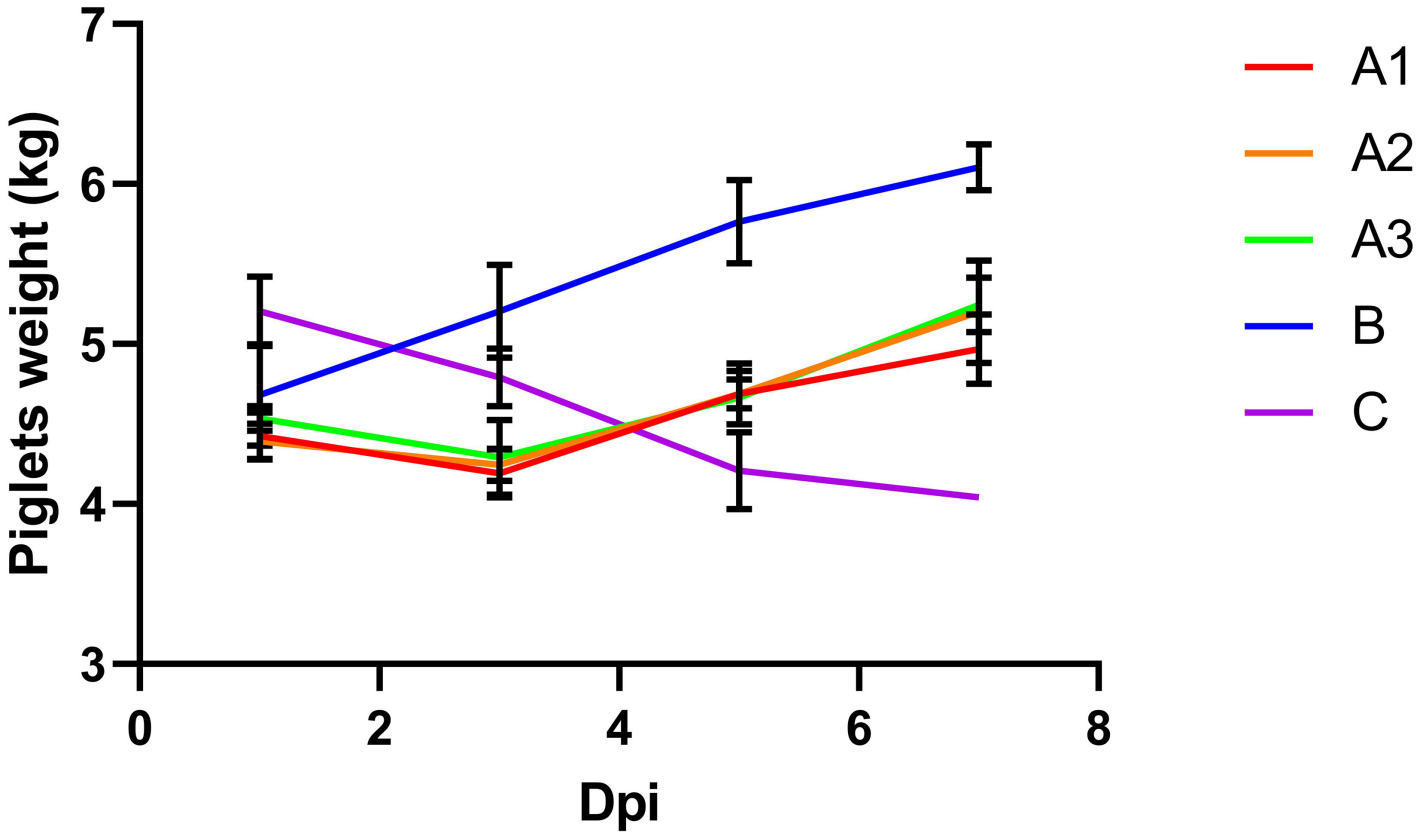
After challenge, group A was treated with 5, 10 and 20 g GML on day 1, 2 and 3, respectively; group B was not challenged or given drugs; group C was not given drugs after challenge, and the weight of piglets in different groups changed with time. Different colors represented different groups. The body weight of piglets in group A decreased from day 1 to day 3 and increased from day 4 to day 7. The weight of piglets in group B increased over time. The weight of piglets in group C decreased over time.

The piglets in group B showed normally body weight gain. The piglets in group C continued to lose weight until death (Figure 1). The piglets in group As lost a slight amount of body weight in the first 3 days after the virus attack. But these piglets started to recover bodyweight gain after the treatment of GML. The results showed that GML was effective in slowing down the weight loss of piglets and subsequently restoring their growth performance.

### Necropsy and histopathological examination were performed

**Figure 2:**
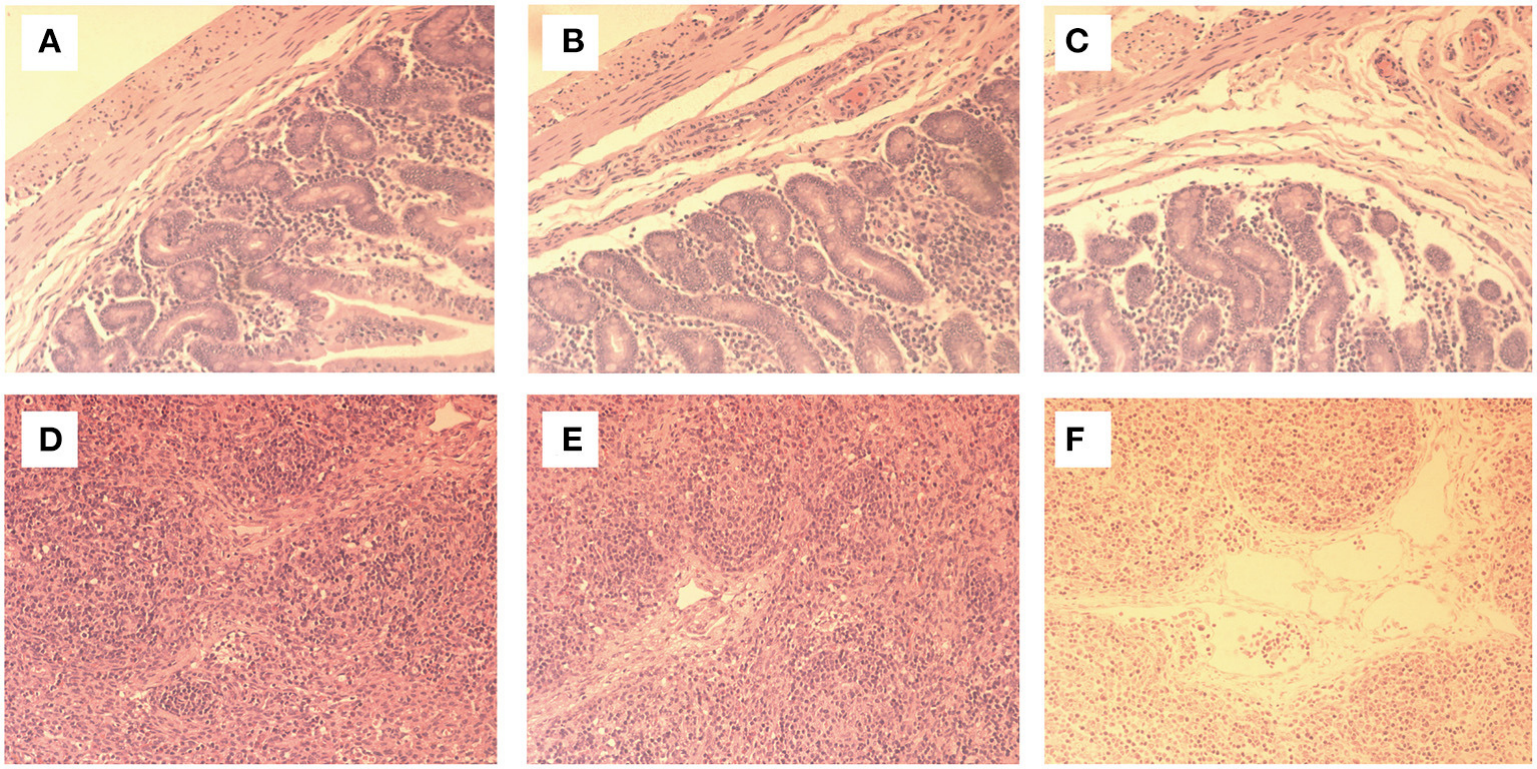
On the 7th day of the experiment, the intestines and mesenteric lymph nodes of the piglets were sectioned and fixed and made into tissue sections for observation. Histopathological examination showed that the morphology of group B was normal and neatly arranged, with intestinal villi, goblet cells and other structures clearly visible **(A)**. The morphology of lymph nodes was normal, without histopathological changes **(D)**. In group A1, the intestinal villi were slightly damaged and the other structures were normal **(B)**. Some lymph node cells were enlarged **(E)**. There were no obvious pathological changes in A2 and A3 groups. In group C, intestinal villi were broken, atrophic and shortened, and some intestinal villi were damaged. Some intestinal epithelial cells were necrotic and exocytic, accompanied by inflammatory cell infiltration **(C)**, increased inflammatory cells in lymph nodes, and tissue edema **(F)**.

The intestinal cavity of the piglets in group C was filled with yellow contents and gas, the intestinal wall was thin, translucent and elastic, and the mesenteric lymph nodes were obviously enlarged, showing typical symptoms of PEDV. Histopathological examination (ileum) showed that there were no obvious abnormalities in intestinal structure and mesenteric lymph nodes between group B and group A. Histopathological examination showed that the intestinal morphology of group B was normal and neatly arranged. The intestinal villi, goblet cells and other structures were clearly visible (Figure 2A), and the lymph nodes were normal (Figure 2D). In group A1, the intestinal villi were slightly damaged, but other structures were normal (Figure 2B). The number of inflammatory cells such as plasma cells and macrophages increased slightly (Figure 2E). No obvious pathological changes were found in groups A2 and A3. In group C, the intestinal villi were ruptured or atrophied, shortened, partially damaged, and some intestinal epithelial cells were necrotic and exfoliated, accompanied by inflammatory cell infiltration (Figure 2C). Lymph node inflammatory cells were increased and the tissue was edematous (Figure 2F).

### PEDV load in intestinal tissues and feces

**Figure 3:**
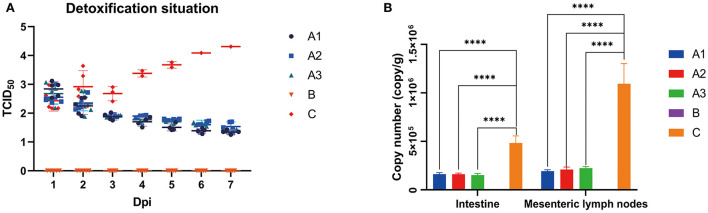
**(A)** shows the copy number of viruses detected by fluorescence quantification in feces of piglets in groups A, B and C on days 1–7. The copy number of viruses in groups A1, A2 and A3 decreased gradually with little difference over time, the copy number of viruses in group B increased gradually over time, and the copy number of viruses in group C was always 0. **(B)** shows the average virus copy number detected by fluorescence quantification in intestinal and mesenteric lymph nodes of piglets in group A, B and C after 7 days of challenge. In the small intestine, there was no significant difference in virus copy number between group A and group B, which was always 0, while the virus copy number in intestinal and mesenteric lymph nodes in group C was larger. *p < 0.05; **p < 0.01; ***p < 0.001; ****p < 0.0001.

As shown in Figure 3A, the fecal viral load in group A peaked on day 1 and then decrease. No PEDV was detected in group B. In group C, the viral load continued to increase to its peak until death; the viral load in both tissues in group A was approximately equal and much lower than that in group C (Figure 3B). The results suggest that GML can reduce the viral load effectively in the feces, intestine and mesenteric lymph nodes of PEDV-infected piglets.

### Cytokine assay

**Figure 4:**
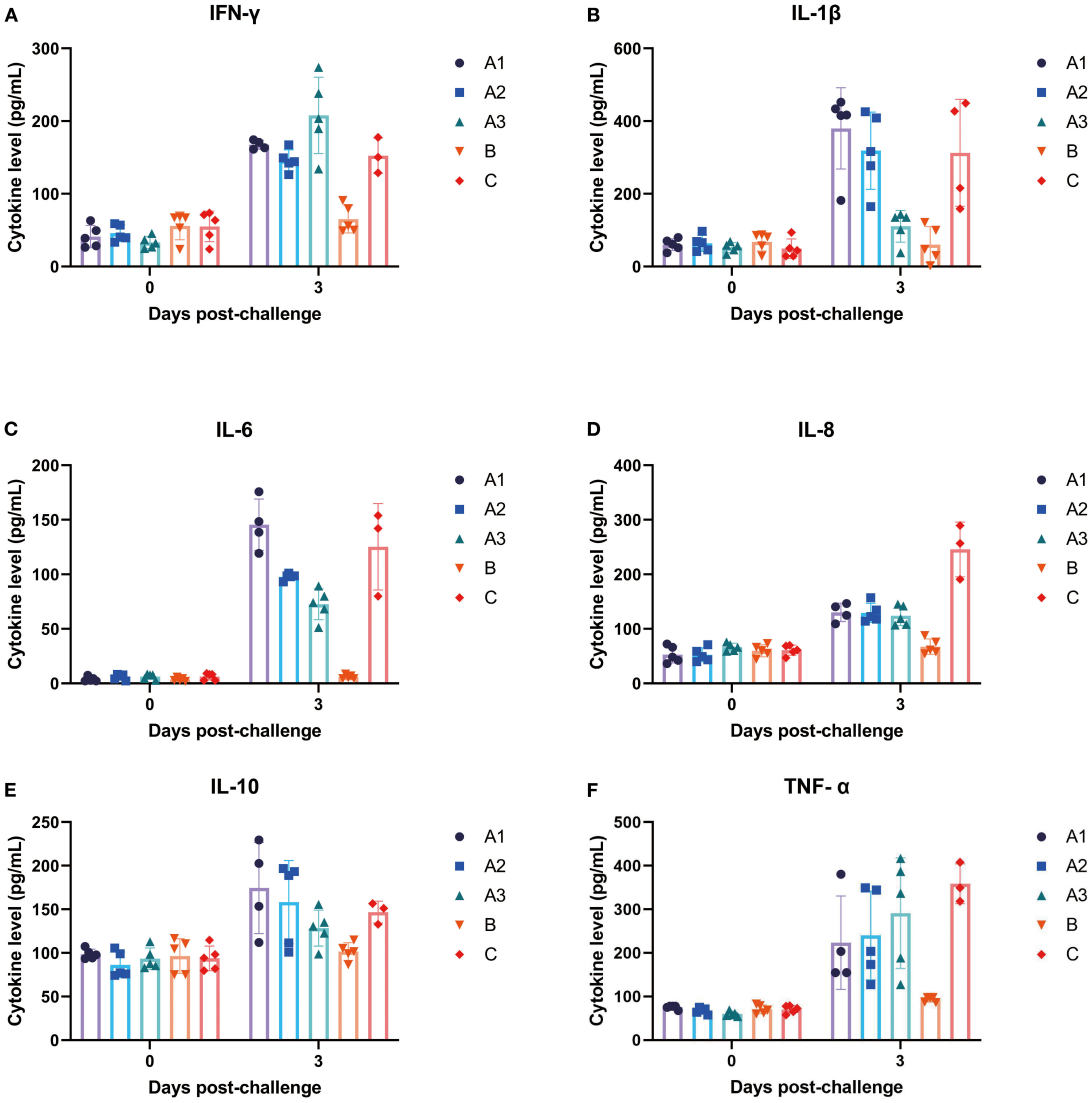
The changes of different cytokines in piglets at day 0–3 were detected. **(A)** IFN-γ, **(B)** IL-1β, **(C)** IL-6, **(D)** IL-8, **(E)** IL-10, and **(F)** TNF-α.

As shown in Figure 4, pro-inflammatory cytokines were significantly increased in Group A and Group C on the third day after infection of PEDV. Compared with group B, the levels of IFN-γ, IL-1β and IL-6 increased significantly in group A, while the dose of GML has a negative correlation with the increasing trend of IL-1β and IL-6. Compared with group C, the levels of IL-8 and TNF-α were significantly lower than group A. GML decreased the levels of IL-1β, IL-6 and IL-10 in a dose dependent manner.

## Discussion

PEDV is an acute, highly contagious and highly lethal virus to pigs, causing huge economic losses to the pig industry. Currently, PEDV is mutating at a rapid rate and is on an outbreak trend, with widespread epidemics in China (10). Existing PEDV vaccines hardly provide effective protection for piglets. Interferons are useful in the early stages of PEDV infection, but the high cost of treatment makes it not a good option (11). Studies indicate that laurate monoglycide has excellent antiviral effect and is a potential anti-PEDV drug (12). At the same time, as a natural active substance, it can be widely used, with non-toxic, harmless, and no residue characteristics. In this study, the results indicated that GML can reduce mortality, weight loss, virus load, pathological injury and inflammation of PEDV infected piglets.

Li et al. (13) showed that the addition of 0.4% α-GML could improve fat metabolism and growth performance of weaned piglets. Lan et al. (14) showed that the addition of 1,000 mg/kg α-GML to the diet significantly increased the average daily weight gain of weaned piglets, indicating that α-GML could improve the growth performance of piglets. Our study also showed that GML was able to slow down the weight loss, restore their growth performance, and increase survival rate for PEDV infected piglets. The results of pathological changes also showed that GML could inhibit PEDV-induced intestinal epithelial cell necrosis.

PEDV infection mediates the body's production of SIgA, which plays an important role in intestinal mucosal immunity. Lin (15) studies have shown that the addition of α-GML to the diet can increase the titer of IgA in the colostrum of sows after vaccination and effectively prevent and control epidemic diarrhea in piglets. In addition, GML can significantly increase the level of SIgA in the ileum of piglets (16). In this trial, the viral load in the feces, intestine and mesenteric lymph nodes of piglets was significantly reduced in the low, medium and high dose groups of GML. This suggests that GML inhibits the proliferation of PEDV *in vivo*, probably because GML can insert into the virus capsule and disrupt the capsule structure of the virus (17), which directly reduces the infection titer of the virus and stimulates the production of SIgA to neutralize the virus, indirectly reducing the transmission and replication of PEDV.

Studies have shown that the addition of 1,000 mg/kg of α-GML to the basal diet significantly increased the relative abundance of the thick-walled phylum, while reducing the number of the phylum Bacteroides and Campylobacter, improving the intestinal structure and reducing the rate of diarrhea in weaned piglets (18). The results showed that the treatment of α-GML significantly increased the relative abundance of thick-walled bacteria and reduced the number of Bacteroides and Campylobacter. Jiang (19) found that the 150 mg/kg GML increased the relative abundance of intestinal butyric acid producing bacteria and the expression of propionic acid and butyric acid, which in turn reduced the permeability. The addition of GML to low protein diets increased the levels of the intestinal tight junction proteins claudin-1, occludin and ZO-1 (16). This is an effective way to ensure the integrity of the intestinal barrier in piglets. In this study, the intestinal morphology of piglets in the high and medium dose groups was intact, which is consistent with the findings of previous studies, indicating that different doses of GML have the ability to promote piglets' resistance to PEDV. We speculate that this may be related to the ability of GML to alter the structure of the intestinal flora, promote the expression of some intestinal tight junction proteins, reduce intestinal permeability and improve the biological and mechanical barriers of the intestinal mucosa.

Zhang et al. (20) showed that GML reduced the production of pro-inflammatory factors such as IL-2, IFN-γ, TNF-α and IL-10 induced by TCR, and the anti-inflammatory effect was dose-dependent within a certain range (18). Also they showed that high concentrations of GML reduced TNF-α levels in the blood of piglets. In the present study, a similar phenomenon was found, with the levels of TNF-α and IL-10 in the blood of piglets in the high-dose treatment group significantly reduced, and the inflammatory cell infiltration was suppressed. This indicated that GML was effective in alleviating PEDV-induced intestinal inflammation, while the elevated levels of IFN-γ were different from the results of previous studies, which may be related to the different levels of activation of the animals' own immune systems or caused by other ingredients in the samples. The effects of the components alone and in combination are unclear, and the existence of synergistic effects needs to be further explored.

## Data availability statement

The original contributions presented in the study are included in the article/supplementary material, further inquiries can be directed to the corresponding authors.

## Ethics statement

The animal study was reviewed and approved by the Sichuan Provincial Laboratory Animal Management Committee [License No: SYXK (chuan) 2019-187]. Written informed consent was obtained from the owners for the participation of their animals in this study.

## Author contributions

ZL, HT, YH, and LZha did the experiment. ZX and JL made important contributions to the analysis and manuscript compilation. HC and XZ conducted data analysis. LZhu and ZL wrote the first draft and revised the manuscript. XS helped with the analysis through constructive discussions. All authors proposed the research concept, directed the research activities, contributed to the article, and approved the submitted version.

## Funding

This study was financially supported by the Sichuan Provincial Department of Science and Technology Rural Area Key R&D Program (Project Number: 2020YFN0147), the Sichuan Province's 14th Five-Year Plan Sichuan Pig Major Science and Technology Project (Project Number: 2021ZDZX0010), the Research and Demonstration of Key Technologies for Prevention and Control of Major Vertically Transmitted Diseases in Breeding Pigs (Project Number: 2022YFN0007).

## Conflict of interest

Author XZ, JL, HC, and YH were employed by Innovation Center of Guangdong Nuacid Biotechnology Co., Ltd. The remaining authors declare that the research was conducted in the absence of any commercial or financial relationships that could be construed as a potential conflict of interest.

## Publisher's note

All claims expressed in this article are solely those of the authors and do not necessarily represent those of their affiliated organizations, or those of the publisher, the editors and the reviewers. Any product that may be evaluated in this article, or claim that may be made by its manufacturer, is not guaranteed or endorsed by the publisher.
